# Optimisation of fermentation conditions for the production of gamma-aminobutyric acid (GABA)-rich soy sauce

**DOI:** 10.1016/j.heliyon.2024.e33147

**Published:** 2024-06-18

**Authors:** Chong Shin Yee, Zul Ilham, Acga Cheng, Muhamad Hafiz Abd Rahim, Siti Hajar-Azhari, Mohd Hafis Yuswan, Nurul Aqilah Mohd Zaini, Anna Reale, Tiziana Di Renzo, Wan Abd Al Qadr Imad Wan-Mohtar

**Affiliations:** a Functional Omics and Bioprocess Development Laboratory, Institute of Biological Sciences, Faculty of Science, Universiti Malaya, 50603, Kuala Lumpur, Malaysia; b Environmental Science and Management Program, Institute of Biological Sciences, Faculty of Science, Universiti Malaya, 50603, Kuala Lumpur, Malaysia; c Faculty of Food Science and Technology, Universiti Putra Malaysia, Serdang, 43400, Selangor, Malaysia; d Halal Products Research Institute, Universiti Putra Malaysia 43400 UPM Serdang, Malaysia; e Department of Food Sciences, Faculty of Science and Technology, Universiti Kebangsaan Malaysia, 43600 UKM Bangi, Selangor, Malaysia; f Institute of Food Sciences, National Research Council, Via Roma 64, 83100, Avellino, Italy

**Keywords:** *Aspergillus oryzae*, *Bacillus cereus*, *Tetragenococcus halophilus*, Response surface methodology (RSM), Sensory evaluation

## Abstract

This study addresses the challenge of enhancing gamma-aminobutyric acid (GABA) content in soy sauce through optimized fermentation condition. Using a multiple starter culture, consisting of *Aspergillus oryzae* strain NSK, *Bacillus cereus* strain KBC and *Tetragenococcus halophilus* strain KBC, the incubation conditions including the percentage of bacterial inoculum (10, 15 and 20 %), pH (3, 5 and 7) and agitation speed (100, 150 and 200 rpm) were optimized through Response Surface Methodology (RSM). Under the optimal conditions (20 % inoculum, pH 7 and stirring at 100 rpm), the multiple starter culture generated 128.69 mg/L of GABA after 7 days and produced 239.08 mg/L of GABA after 4 weeks of fermentation, which is 36 % higher than under non-optimized conditions (153.48 mg/L). Furthermore, sensory analysis revealed high consumer acceptance of the fermented soy sauce than the control (soy sauce without any treatment and additional bacteria) and commercial soy sauce. Consumers indicated that the starter culture offered an improved umami taste and reduced bitter, sour and salty flavours compared to the commercial product. Under optimal fermentation conditions determined by RSM statistical analysis, the multiple starter culture is able to produce high levels of GABA and is more likely to be accepted by consumers. The findings of this research have the potential to impact the food sector by offering a functional soy sauce with added health benefits and also being well-received by consumers.

## Highlights

• Response Surface Methodology to optimise fermented GABA-rich soy sauce production.
• Optimized conditions and selected microbial starter to produce GABA-rich soy sauce.
• GABA-enriched soy sauce has a high level of consumer acceptance.

## 1 Introduction

The origins of soy sauce, a fermented condiment, date back to ancient China. It is a light-brown salty liquid made from fermented soybeans, wheat, salt, and water. It has an umami-rich flavour profile and is a staple food in many cuisines worldwide [1]. Soy sauce offers a rich, savoury flavour that can help replace the umami flavour typically of meat and other animal products. It is also a key component of many vegan and vegetarian recipes [2]. In fermented soy sauce a “starter culture”, containing a mixture of different microbial species, is used to trigger the degradation of macromolecules in the raw materials [3]. Free amino acids, nucleotides and small peptides are the compounds mainly linked to the distinctive aromas of soy sauce, including malty, caramel, cooked potato, floral, alcoholic, sour, smoky, seasoning and fruity notes [3,4].

Soy sauce is characterised by five primary flavours (sweet, salty, sour, bitter and umami). The harmonious combination and delicate balance of these tastes explain why it is used as universal condiment. Each component's aroma and taste threshold are crucial for determining the sensory impact.

The fermentation process is used not only to improve the quality characteristics of the product and extend its shelf life, but increasingly also to generate bioactive molecules that offer benefits to the consumer [5,6]. Gamma-aminobutyric acid, or GABA, is one of the main neurotransmitters claimed to have a calming effect on the brain and nervous system. In the motor system, GABA pre-synaptically inhibits the primary afferent fibers and plays a role in the formation of motor neuron inhibition during post-synaptic processes in the central nervous system (CNS). The CNS depends on amino acid neurotransmitters for proper operation. These quick-acting neurotransmitters produce responses in milliseconds that are crucial for normal brain function as well as the prevention of neurological disorders [7].

Due to its health benefits, GABA has recently been used as a functional ingredient in various foods [8]. GABA is contained in low amounts in many foods, however, directly adding GABA to food is deemed unsafe and is known as unnatural [9]. Therefore, fermentation by different microbial species able to produce this compound can be considered a promising possibility to enhance the nutritional, sensory and functional properties of specific fermented foods [[10], [11], [12]]. As reported by Gopikrishna et al. (2021) [13] fermented soy products (soy sauce, soy paste, tempeh, natto, sufu, soy nuggets, and soy yogurt) contain species belonging to the genus *Bacillus* (*B. amyloliquefaciens, B. cereus, B. circulans, B. licheniformis, B. sphaericus, B. subtilis,* and *B. thuringiensis),* lactic acid bacteria (LAB), filamentous fungi, yeasts, *Aspergillus*, *Torulopsis*, *Zygosaccharomyces* and *Rhizopus*. These microorganisms can produce bioactive compounds during fermentation that have beneficial impacts in improving human health. With regard to soy sauce, a study by Ab Kadir et al. [14], showed that four strains of *Aspergillus oryzeae* isolated from soy sauce *koji* were able to produce GABA. In particular, the *Aspergillus oryzae* NSK strain showed the highest GABA values (194 mg/L) and the authors suggested its possible use as a starter culture for soy sauce production. In addition, other authors [11,12] isolated from moromi and successfully identified a strain belonging to the species *Bacillus cereus* (*Bacillus cereus* KBC) and *Tetragenococcus halophilus* (*Tetragenococcus halophilus* strain KBC) with high GABA production.

Therefore, in the present study, using Response Surface Methodology (RSM), the production of GABA by a multiple starter culture consisting of *Aspergillus oryzae* strain NSK, *Bacillus cereus* strain KBC and *Tetragenococcus halophilus* strain KBC in soy sauce was optimized. Furthermore, consumer acceptance of the GABA-enriched soy sauce was evaluated.

## 2 Material and methods

### 2.1 Microorganisms and cultural conditions

*Aspergillus oryzae* strain NSK (*A. oryzae*), *Bacillus cereus* strain KBC (*B. cereus*) and *Tetragenococcus halophilus* strain KBC (*T. halophilus*) were obtained from the Functional Omics and Bioprocess Development Laboratory, Institute of Biological Sciences, Faculty of Science, Universiti Malaya.

Strains belonging to *Bacillus* and *Tetragenococcus* species were successfully isolated and identified from a soy sauce company (Kwong Bee Chun Soy Sauce Sdn. Bhd.) located at Kamunting, Perak, Malaysia [11,12]. The pure culture of *koji* spores belonging to *Aspergillus* species was isolated using the single spore isolation technique with some modifications [15]. The spores were then processed into a powder form for future use. *B. cereus* and *T. halophilus* were cultured for 72 h at 30 °C in de Man, Rogosa, Sharpe (MRS) broth (69964-500G, Sigma-Aldrich, Dorset, UK).

*B. cereus* was cultured under aerobic conditions, while *T. halophilus* was cultured under anaerobic conditions in 5 % NaCl (food grade) and 0.5 % (w/v) of CaCO_3_ (Bendosen Laboratory Chemicals, Bendosen, Norway) using an anaerobic jar equipped with *Anaerocult* A (Merck, Darmstadt, Germany) [11].

The chemicals and reagents used in the study were of analytical grade and were purchased from Sigma-Aldrich Chemistry (Madrid, Spain).

### 2.2 *Koji* fermentation

The soybeans were soaked overnight in water and then cooked for 3–6 h at 100–110 °C until soft. The wheat grains were roasted, ground, and combined with the cooked beans and starter culture, which was the *koji* mold. The soybeans, wheat, and starter culture (*Aspergillus oryzae* strain NSK) ratios were 50, 25 and 0.25 g, respectively according to the company recipe. After mixing all the ingredients, the *koji* was incubated at room temperature around 30 °C for one week to allow the starter to grow and sporulate. A greenish-yellow mash was observed because of mold growth and sporulation.

### 2.3 Moromi fermentation

**Fig. 1:**
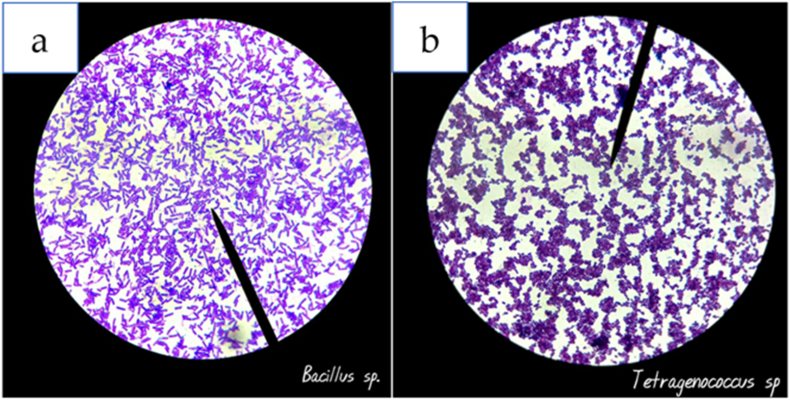
**(a)***Bacillus cereus* strain KBC and **(b)***Tetragenococcus halophilus* strain KBC (Author's personal collection) observation by light microscope Olympus BX40.

The mature *koji* was added to a 20 % brine solution to create *moromi* mash. *B. cereus* strain KBC (Fig. 1a) and *T. halophilus* strain KBC (Fig. 1 b) were added into the *moromi* mash to allow *moromi* fermentation. In detail, both *B. cereus* and *T. halophilus* from the agar slant were spread on MRS agar plates and incubated at 37 °C for 2 days to allow the bacteria to grow and reproduce. After 2 days of inoculum, a single colony was transferred into 150 mL of MRS broth in a 200 mL shake flask and incubated at 37 °C for further 3 days.

Then, a mixed culture (10^3^ cfu/mL) consisting of *B. cereus* strain KBC and *T. halophilus* strain KBC in a 1:1 ratio was obtained. This mixed culture was added to the *moromi* mash and incubated aerobically at 37 °C under different conditions (10, 15 and 20 % inoculum percentage, initial pH 3, 5 and 7, and 100, 150 and 200 rpm agitation on a shaker (KS 4000 ic, IKA®-Werke, Staufen, Germany). Naturally fermented *moromi* (non-inoculated, pH 6, without agitation) was used as control sample. In the *moromi* fermentation stage, three different types of analysis were done (Table 1). Firstly, to obtain the optimized *moromi* fermentation condition (inoculum percentage, pH and agitation speed) for GABA production, an experiment was designed using Response Surface Methodology (RSM).

**Table 1:** Fermentation time of each analysis.

Type of Analysis	Duration of *moromi* fermentation (days)
Response Surface Methodology (RSM)	7
GABA production under optimized and non-optimized condition	28
Sensory Analysis	28

Accordingly, Table 2 and Table 3 summarized 20 experimental runs during *moromi* fermentation using 250 mL Erlenmeyer flask for 7 days. Secondly, to evaluate the GABA enhancement by the multiple starter culture, the duration of *moromi* fermentation was 28 days under both non-optimized and optimized conditions. Samples were collected at 7-day intervals. Lastly, to evaluate consumer acceptance, sensory analysis was done using both non-optimized and optimized *moromi* incubated for 28 days. The obtained soy sauce was then compared with the commercial soy sauce for its sensory attribute.

**Table 2:** The selected variables range and levels input for optimisation study.

Variables		Range and Levels	
	−1	0	1
Inoculum percentage (%)	10	15	20
pH	3	5	7
Agitation speed (rpm)	100	150	200

**Table 3:** Experimental runs of three variables according to Central Composite Design (CCD). Experimental and predicted values of gamma-aminobutyric acid (GABA) production (mg L^−1^) obtained using the multiple starter culture consisting of *Aspergillus oryzae* strain NSK, *Bacillus cereus* strain KBC and *Tetragenococcus halophilus* strain KBC.

Run No.	Variables			Response	
	Inoculum percentage (%)	pH	Agitation (rpm)	GABA (mg/L)	
				Experimental	Predicted
1	15	5	150	93.24	83.20
2	10	7	100	85.32	87.17
3	15	5	150	79.32	83.20
4	10	5	150	83.44	80.64
5	10	3	200	55.55	59.47
6	10	3	100	83.68	80.98
7	15	7	150	81.67	80.78
8	20	3	100	95.67	96.21
9	15	5	200	67.42	62.40
10	15	5	150	82.24	83.20
11	20	3	200	51.41	49.82
12	15	3	150	68.56	68.39
13	20	7	200	65.44	68.40
14	15	5	150	80.74	83.20
15	10	7	200	55.69	55.42
16	15	5	100	97.51	101.47
17	20	5	150	93.00	94.75
18	15	5	150	82.09	83.20
19	15	5	150	79.49	83.20
20	20	7	100	128.69	125.03

### 2.4 GABA detection and quantification

The method of Yang et al. [16] with slight modifications was used to determine the GABA content of the soy sauce samples. The soy sauce was first filtered through Whatman No.2 filter paper, and then filtered through a 0.22 μm pore-size nylon filter (Fisher Scientific Bishop Meadow, UK). High-performance liquid chromatogram (HPLC) was used to analyse the filtered sample.

For the HPLC analysis, 1 mL of the analyte was placed in an HPLC autosampler glass vial. A mobile phase was prepared with 60 % solution A (aqueous solution of 8.205 g sodium acetate, 0.5 mL triethylamine and 0.7 mL acetic acid in 1000 mL) adjusted to pH 5.8, 28 % solution B (deionized water) and 12 % solution C (acetonitrile). The separation was performed using a Shimadzu LC 20AT apparatus, consisting of a pump system, a CT0-10ASVP model oven with a 20-μL injection loop injector and an SPD-M20A PDA detector in conjuction with a DELL model DELL Optiplex integrator. For separation purposes, a Hypersil Gold C-18 column (250 × 4.6 mm I.D., particle percentage 5 μm; Thermo Scientific, Meadow, UK) was used. The mobile phase for gradient elution was pumped at 0.6 mL/min at 25 °C, and detection was monitored at 254 nm. The GABA content of the sample was determined by comparing the peak of the graph to the GABA standard curve [17].

### 2.5 Optimisation of moromi fermentation conditions by RSM

Based on the preliminary studies, inoculum percentage, pH and agitation speed affect GABA production in soy sauce. According to Wan-Mohtar et al. [17], the selected independent variables are able to influence the multiple starter culture (*A. oryzae*, *B. cereus*, and *T. halophilus*) to produce a high concentration of GABA. To obtain the optimal GABA production, the independent variables were designed using RSM with a Central Composite Design (CCD). The range and the levels of the independent variables are given in Table 2.

Twenty experiments were generated and three level face centered cubic design was implemented. The investigated independent parameters were inoculum percentage (10, 15 and 20 %), pH (3, 5 and 7) and agitation speed (100, 150 and 200 rpm). The experimental design runs were prepared in a 250 mL Erlenmeyer flask and fermentation was employed under controlled condition. The data was collected included both predicted and actual responses regarding the GABA production.

Experimental results were fitted with a second order polynomial equation by multiple regression analysis. The quadratic mode for predicting the optimal point was expressed according to Eq. (1), where Y represents the response variable (GABA content), β_i_ are the regression coefficients and X_1_, X_2_ and X_3_ represent the independent variables.

(1) Y = β0 + β_1 × 1_ + β_2_X_2_ + β_3_X_3_ + β_1_X_1_^2^ + β_1_β_2_X_1_X_2_ + β_2_X_2_^2^ + β_1_β_2_β_3_X_1_X_2_X_3_ + β_3_X_3_^2^

Design Expert 11.0 software (Version 11, Godward Street NE, Suite 6400, Minneapolis, MN, USA) was used for the experimental design and regression analysis of the experimental data. The effects of linear, quadratic and interactive terms of the independent variables on the GABA production were evaluated by the model. The quality of the fitted polynomial model was statistically checked by the magnitude of the coefficient of determination R^2^ and its statistical significance was checked by the *F*-test analysis of variance (ANOVA). The coefficients of the response surface were evaluated using the student *t*-test.

### 2.6 Model validation using verified conditions on GABA response

RSM-optimized incubation conditions, including 20 % bacterial inoculum, pH 7 and 100 rpm agitation speed, were used to perform a validation test of model. Experiments were carried out in triplicate using 250 mL Erlenmeyer flasks and the *moromi* fermentation lasted 28 days. Naturally fermented *moromi* (non-inoculated, pH 6, without agitation) was used as control sample.

### 2.7 Sensory evaluation

**Fig. 2:**
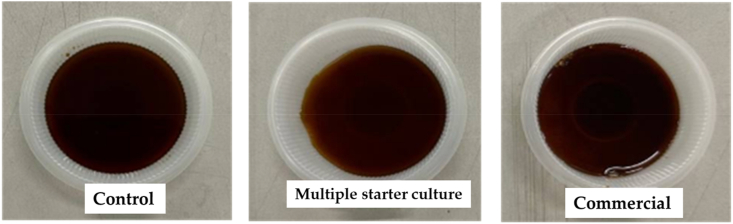
Soy sauce samples used for sensory analysis: multiple starter culture, commercial and control.

Sensory evaluation and acceptability tests were used to define the sensory profiles and the level of pleasantness of the different samples of soy sauce. Sensory evaluation includes assessment of taste and overall product acceptance. The soy sauce samples, after fermentation and heat treatment, was assessed by a panel of 300 untrained consumers (males and females, aged between 18 and 60 years, students and staffs) from Universiti Malaya, Kuala Lumpur, Malaysia. Tests were conducted in triplicate, following a previous method [17] with some modifications. Each panelist was given three coded samples 815, 701 and 525, representing the multiple starter culture, commercial and control, respectively (Fig. 2), to be tested with a plain rice ball.

Six common sensory attributes were evaluated: sweet, umami, salty, bitter, sour, and astringent and the overall acceptability of the consumer were evaluated by using a 10-cm line scale with 0 denoting none and 9 denoting extremely strong. First, 10 mL of each soy sauce sample was placed into a plastic sauce plate, and a small rice ball was served to the panelists to taste together with the sauce. After each evaluation, unsalted crackers and mineral water were served as palate cleansers to the consumers.

### 2.8 Statistical analyses

Each experiment was run in triplicate for the RSM, and the findings are presented as means. Using Design Expert 11.0 software, analysis of variance (ANOVA) was utilized to evaluate significant differences between variables with a p -value <0.05.

The statistical data from the sensory analysis were analyzed using IBM SPSS Statistics software (version 29; IBM, Chicago, Illinois, USA). Each sample was tested in triplicate. The results are presented as means ± standard deviation. Tukey's test with a 95 % confidence level was performed to determine whether there was a significant difference between samples using one-way analysis of variance (ANOVA) with the Tukey Post Hoc test. The result was considered statistically significant if the *p*-value is less than 0.05.

## 3 Results and discussion

### 3.1 Optimisation of moromi fermentation conditions for GABA production

RSM is a statistical approach for determining the best combination of input variables to optimise a desired outcome. In this study, bacterial inoculum percentage (10, 15 and 20 %), pH (3, 5 and 7) and agitation speed (100, 150 and 200 rpm) were chosen as the variables, and the desired outcome or the response was GABA production. Table 3 shows 20 runs of experiments generated using CCD design in the Design Expert 11.0 software.

The ANOVA for GABA production using the multiple starter culture (*A. oryzae*, *B. cereus*, and *T. halophilus*) is presented in Table 4. The coefficient of regression for this model is R^2^ = 0.9586, which explains that 95.86 % of the model response's variability. R^2^ is a measure used to indicate how well a regression model fits the data, with a higher value indicating a better fit [18].

GABA = 47.6009–3.07498 × Inoculum Percentage+ 19.98939 × pH + 0.262322 × Agitation +0.565875 × Inoculum Percentage × pH −0.024875 × Inoculum Percentage × Agitation – 0.025613 × pH × Agitation +0.179582 × Inoculum Percentage^2^ – 2.15386 × pH^2^ - 0.000506 × Agitation^2^

**Table 4:** Analysis of variance (ANOVA) for the experimental results of the CCD quadratic model for GABA production using the multiple starter culture.

Source	Sum of Squares	DF	Mean Square	*F* Value	Prob > *F*	
Model	5594.86	9	621.65	25.74	<0.0001*	Significant
A- Inoculum Percentage	497.45	1	497.45	20.59	0.0011*	Significant
B- pH	383.66	1	383.66	15.88	0.0026*	Significant
C- Agitation	3816.55	1	3816.55	158.01	<0.0001*	Significant
AB	256.17	1	256.17	10.61	0.0086	Significant
AC	309.38	1	309.38	12.81	0.005	Significant
BC	52.48	1	52.48	2.17	0.1713	
A^2^	55.43	1	55.43	2.29	0.1608	
B^2^	204.12	1	204.12	8.45	0.0156	Significant
C^2^	4.4	1	4.4	0.1823	0.6784	
Residual	241.55	10	24.15			
Lack of Fit	104.44	5	20.89	0.7618	0.6137	Not Significant
Pure Error	137.1	5	27.42			
Cor Total	5836.4	19				
Std.Dev = 4.91		R^2^ = 0.9586			Adeq Precision = 21.6431	
Mean = 80.51		Adjusted R^2^ = 0.9214				

In addition, the adjusted coefficient determination value (Adj.R^2^ = 0.9214) indicates that the model was significant and that the adjusted R^2^ value was within a reasonable range of agreement with the predicted R^2^ value. This adjusted R^2^ ensures that the additional variables contribute meaningfully to the model's explanatory power, making it a valuable tool for model evaluation, especially in cases involving multiple independent variables [19]. Besides, the *p*-value <0.0001 indicates that the model is significant. This model has a non-significant Lack of Fit with a *p*-value of 0.6137 (*p* > 0.05). With all these values, the overall quality and the reliability of this model in predicting the GABA production yield is highly acceptable [20].

According to Table 4, the effects of the Inoculum Percentage (A), initial pH (B), agitation (C) and quadratic terms (AB, AC, B^2^) on the production of GABA are significant (*p* < 0.05).

This is because the bacteria inoculum percentage has a crucial impact in the production of GABA. This parameter is critical in determining the initial concentration of GABA-producing bacteria, which is then added into the soy sauce *moromi* mash. An increase in inoculum percentage has the potential to decrease the lag phase, hence accelerating GABA production. However, an extreme high bacteria inoculum percentage leads to intensified competition among bacteria for essential nutrients and has the potential to reduce or halt GABA production. In fact, also other authors highlighted that a higher inoculum size (∼10^5^ cfu/mL) could trigger bacterial population growth resulting in nutrient depletion and production of toxic metabolites [21]. Consequently, the bacterial population could enter the death phase earlier resulting in lower GABA production. Hence, finding an appropriate equilibrium by optimizing the bacteria inoculum percentage is necessary to achieve optimal GABA yields and cost-effective production.

Furthermore, the effects of pH regulation on the production of GABA in Table 4 are also significant (*p* < 0.05). This is due to the fact that pH is one of the critical factors influencing GABA production since it influence the enzymatic activity, specifically the Glutamate Decarboxylase (GAD) enzyme [[22], [23], [24]] that plays a pivotal role in the α-decarboxylation reaction of the l-glutamate. This irreversible enzymatic reaction leads to a significant transformation, forming the important neurotransmitter GABA [25]. In this reaction, GAD act as a catalyst, facilitating the removal of a carboxyl group (COOH) from the l-glutamate molecule. Removing the carboxyl group is a key step in the conversion process, and because of this α-decarboxylation, the l-glutamate molecule is structurally altered, resulting in GABA [26]. GAD enzyme works well only in a specific pH range [27,28] and consequently, it is crucial to maintain pH levels within this ideal range to facilitate efficient production of GABA. The pH level also has a considerable impact on the development and metabolic activities of GABA-producing strains. Variations from the ideal pH level can affect bacterial multiplication, hence GABA production [24,29].

Different authors showed that temperature, pH and initial glutamate concentration had a significant effect on the cell growth of *Lb. brevis* NCL912 and the GABA production [29]. Not only that, another research also found that the ideal pH for high GABA production by *Bacillus cereus* strain KBC is pH 7.0 [12]. In short, the impact of the pH factor on GABA synthesis is strain-specific, and the optimum pH for achieving maximum synthesis of GABA is reliant upon the species and varies greatly.

Besides, according to Table 4, the effects of the agitation (C) on the production of GABA are also significant (*p* < 0.05). Agitation acts as a crucial catalyst for promoting optimal production in the fermentation process. The process of mechanically mixing or swirling the culture media facilitates the uniform dispersion of nutrients, oxygen, and vital components, hence promoting microbial proliferation and metabolic activities [30]. Nevertheless, it is important to strike a balance, as excessive agitation can damage the bacterial cells, thereby disrupting the fermentation process. These factors are key to achieving optimal GABA yields and ensuring the overall effectiveness of the fermentation procedure.

**Fig. 3:**
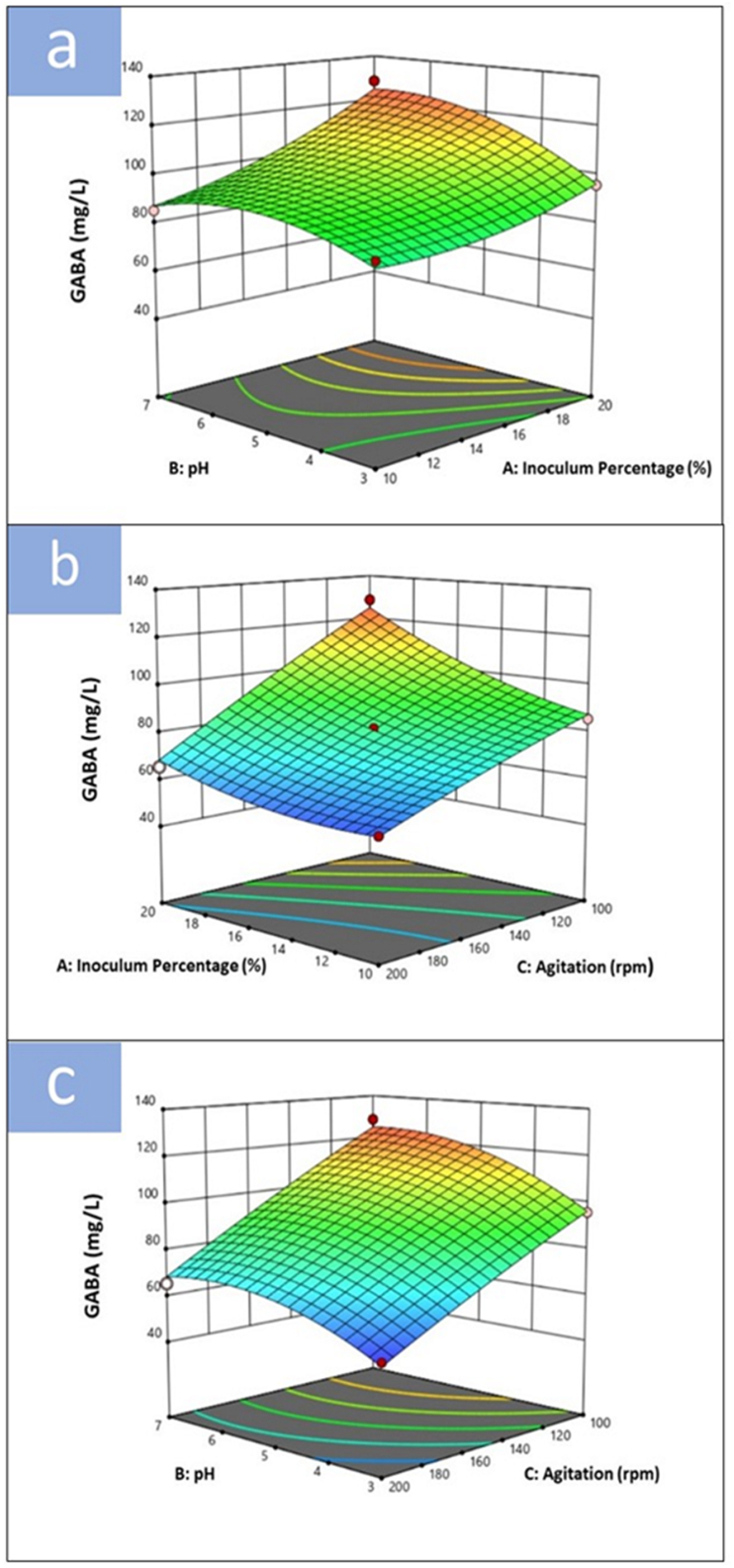
Response surface curve (3D plot) of GABA production from the multiple starter culture consisting of *Aspergillus oryzae* strain NSK, *Bacillus cereus* strain KBC and *Tetragenococcus halophilus* strain KBC displaying the interaction between (a) pH and inoculum percentage, (b) inoculum percentage and agitation speed and (c) pH and agitation speed.

The three-dimensional (3D) graphs illustrate that various quadratic factor combinations have a positive effect on GABA yield (Fig. 3). The effects of pH (B) and inoculum percentage (A); agitation (C) and inoculum percentage (A); pH (B) and agitation (C) on the production of GABA are shown in Fig. 3a, b, and 3c, respectively.

This result shows that these factors set in particular quantity or circumstances, promote GABA production. In short, at 20 % bacteria inoculum percentage, pH 7, and 100 rpm, the maximum GABA concentration 128.69 mg/L can be obtained in 7 days.

### 3.2 Verification of optimized condition

The effectiveness of this model was confirmed by comparing and assessing the predicted GABA yield under statistically optimal conditions (Table 5). A validation test was executed in triplicate using 250 mL shake flasks under the optimal conditions of 20 % bacterial inoculum, pH 7 and 100 rpm. The model's predictive capability was validated using Eq. 2. The experimental value for GABA production was 128.69 mg/L, whereas the expected value was 125.03 mg/L. The predicted value was 3.66 mg/L lower than the experimental value, and the difference between the values was only 1.03 %. Given that the average deviation is below 15 %, it can be concluded that this model is valid [31].

**Table 5:** Validation of model using verified condition on GABA response.

Dependent Variable	Response	
	Predicted GABA (mg/L)	Experimental GABA (mg/L)
GABA	125.03	128.69 ± 8.605

### 3.3 GABA production by microbial starter composed by *A. oryzae*, *B. cereus* and *T. halophilus*

**Fig. 4:**
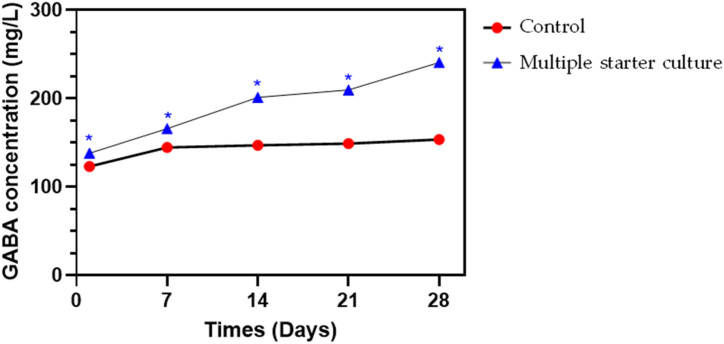
The comparison of GABA production yield by the multiple starter culture (under optimized condition: 20 % bacteria inoculum percentage, pH 7, 100 rpm) and control sample during 28 days of fermentation.

The GABA production from the multiple starter culture (under optimized condition: 20 % bacteria inoculum percentage, pH 7, 100 rpm) and control (without any treatment and inoculation) was monitored by HPLC analysis at 7-day intervals for 28 days. The GABA concentrations produced over time for both the multiple starter culture and control are illustrated in Fig. 4.

Overall, the multiple starter culture exhibited higher GABA production than the control throughout the monitoring period. This indicates that these additional microorganisms have a positive and ongoing effect on the GABA production, causing it to increase steadily. This is consistent with some recent studies which found that the inclusion of microbes does help in boosting the GABA production in soy sauce [11,12,14]. On the other hand, the control, which did not receive additional microbes, initially experienced a slight increase, but quickly levelled off. Over time, the values for this sample remained relatively stable with only minor fluctuations. The observed phenomena may not be attributed to the raised levels of gad gene expression in response to the stress circumstance such as acidic environments and starvation [27,32].

Lactic acid bacteria (LAB) has extensive applications in various fermented food products, such as tempeh, kimchi, yoghurt, cheese, sourdough and soy sauce, with functional and probiotic properties [[33], [34], [35]]. Reports have found that the LAB from fermented food could generate GABA by utilising glutamic acid as a substrate [7]. Recent studies [11,12] have suggested that *B. cereus* and *T. halophilus* are potential GABA-producing strains that can be used in GABA-enriched soy sauce production, and this study validates that the inclusion of supplementary bacteria (*B. cereus* and *T. halophilus*) can effectively amplify GABA production during the soy sauce fermentation process.

### 3.4 Sensory analysis

**Fig. 5:**
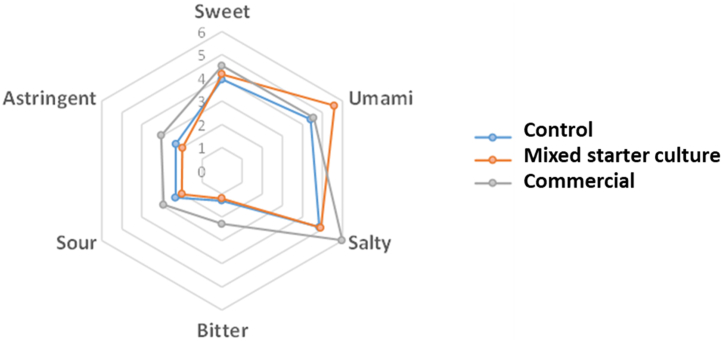
The spider diagram illustrates a descriptive sensory analysis of six taste (sweet, umami, salty, bitter, sour, and astringent) on three different soy sauce samples (Control, mixed starter culture and commercial sample).

Sensory analysis is a systematic and scientific method used to analyse and study the sensory attributes of products or materials, specifically in the food and beverage industry and could be an important tool to develop novel food [36,37]. In this study, six attributes were selected to examine soy sauce sensory profile: sweet, umami, salty, bitter, sour, and astringent. Fig. 5 visually presents average scores for the six attributes across all soy sauce samples. The sensory study included three formulations: control, multiple starter culture and commercial soy sauce (Table 6). The results showed that there were significant differences among the soy sauce samples for the six attributes evaluated (p < 0.05).

**Table 6:** 10-cm line scale for the six tastes (sweet, umami, salty, bitter, sour and astringent) of soy sauce samples (0 = none, 1 = extremely weak, 2 = very weak, 3 = weak, 4 = moderately weak, 5 = moderately, 6 = moderately strong, 7 = strong, 8 = very strong and 9 = extremely strong).

Sample	Preference scores for taste					
	Sweet	Umami	Salty	Bitter	Sour	Astringent
Control	3.93 ± 0.129^a^	4.42 ± 0.338^c^	4.85 ± 0.118^e^	1.29 ± 0.108^g^	2.32 ± 0.124^i^	2.31 ± 0.131^k^
Multiple starter culture	4.15 ± 0.134^ab^	5.59 ± 0.127^d^*	4.91 ± 0.125^e^	1.19 ± 0.110^g^	2.01 ± 0.126^i^	1.98 ± 0.140^k^
Commercial	4.52 ± 0.155^b^*	4.56 ± 0.142^c^	5.98 ± 0.125^f^*	2.29 ± 0.123^h^*	2.92 ± 0.156^j^*	3.04 ± 0.162^l^*

Means ± standard deviations of triplicate independent experiments are shown. Within each column overall means with different superscript letters are significantly different (*p* < 0.05). The symbol asterisk (*) shows the highest mean value.

A significant difference in sweetness was observed between the control and commercial samples (p < 0.05). The commercial sample had the highest mean score of 4.52, followed by the soy sauce obtaining by using multiple starter culture at 4.15 and the control at 3.93. Notably, no significant difference was found between the starter culture-commercial and starter culture-control comparisons, indicating the compatibility of the multiple starter culture with the commercial soy sauce in terms of sweetness.

Regarding umami, the multiple starter culture showed the highest mean score of 5.59, followed by the commercial at 4.56 and the control at 4.42. Significant differences emerged between the starter culture and commercial and control samples (p < 0.05). As one of the key flavour profiles, umami is significantly important for soy sauce flavour. The term ‘umami’, originating from the Japanese language, denotes a sensation of deliciousness [38]. Thus, the higher the umami taste, the better the soy sauce will taste.

Furthermore, the commercial sample had the highest mean scores for salty (5.98), bitter (2.29), sour (2.92) and astringent (3.04) attributes. However, no significant difference was found between the starter culture and control. Intriguingly, a significant difference was observed between the commercial-starter culture and commercial-control comparisons (p < 0.05). Individual soy sauces possess distinct qualities. The observed variability can be attributed to the complicated production process or the fermentation time [3]. Additionally, the commercial soy sauce fermented for three months instead of one month for the starter culture and control in this study. Thus, this might be why the commercial soy sauce had stronger saltiness, bitterness, sourness and astringency attributes than the multiple starter culture and control [39].

**Fig. 6:**
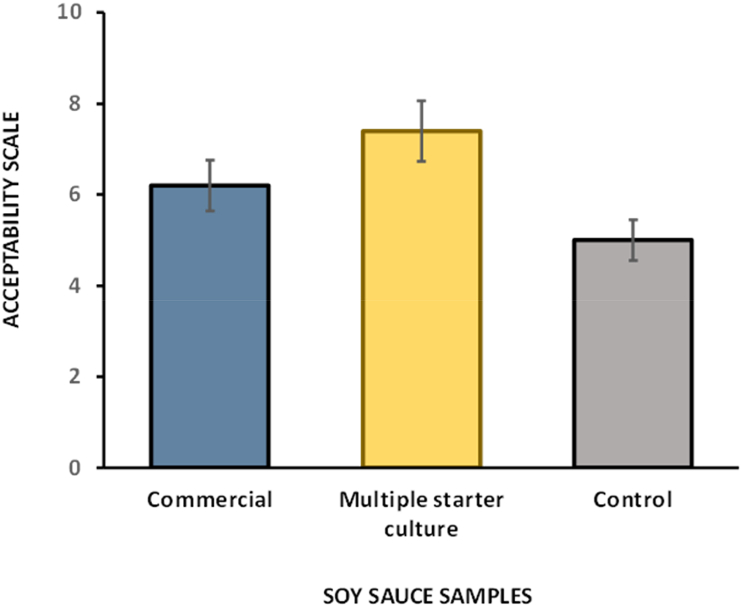
Mean scores for overall acceptability for all the soy sauce samples for 300 panellists.

The multiple starter culture also showed higher overall acceptability compared to the control and commercial soy sauces (Fig. 6). This may be due to the high amount of umami flavour in the multiple starter culture. Moreover, the starter culture had a sweetness (4.15) comparable with commercial soy sauce (4.52). In addition, the starter culture had lower saltiness, bitterness, sourness and astringency attributes than the commercial soy sauce, which was found to be more palatable for consumers.

The global market offers a wide array of contrasting soy sauce products due to distinct production methods, which can be either microbial or chemical synthesis, various microbe cultures introduced during the fermentation, and ageing time. It is well known that each of these soy sauce varieties has a distinctive flavour profile [1]. Soy sauce contains various non-volatile chemicals, mostly free amino acids, 5′-nucleotides, short peptides, soluble saccharides and polyols. Non-volatile compounds play a significant role as flavour molecules, particularly associated with fundamental tastes of sweetness, saltiness, sourness, bitterness and umami [40].

Based on the obtained results, the soy sauce obtaining by using multiple starter culture received a level of acceptance comparable to that of commercial soy sauce by the panellists, and it had a better umami taste score of 5.59 in the sensory test than the commercial soy sauce, which only had a score of 4.56. The starter culture also had less salt, bitterness and sourness than the commercial soy sauce. This study found that the consumer should have a higher acceptability of the soy sauce obtaining by using multiple starter culture than the commercial soy sauce.

## 4 Conclusion

The *A. oryzae*, *B. cereus* and *T. halophilus* multiple starter culture can produce a high GABA amount under optimal fermentation conditions, which were statistically analyzed using RSM in the present study. The findings showed that the bacteria inoculum percentage (A), pH (B) and agitation (C) significant affect GABA production. Under optimized conditions, such as 20 % bacterial inoculum percentage, pH 7 and 100 rpm, the GABA production can increase from 137.98 mg/L to 239.08 mg/L compared to the control from 122.91 mg/L to 153.48 mg/L after 4 weeks of incubation. Additionally, the soy sauce obtained using the multiple starter culture received greater acceptability than both the control and commercial variants. Therefore, based on these findings, we conclude that the multiple starter culture used for the production of soy sauce is able to produce higher GABA when compare to the control and is comparable, if not superior, to the commercial soy sauce in terms of its taste sensory attributes. Considering the importance of microbial fermentation to increase GABA content, optimisation of starter culture conditions is of great interest because it may offer concrete opportunities for the design of new functional foods for consumers seeking natural ways to improve their daily diets with substances, such as GABA, that can reduce mood disorders such as anxiety, depression, insomnia, irritability and restlessness.

## Declarations

### Ethics statement

The sensory evaluation of the soy sauce samples was carried out in accordance with established ethical guidelines and all participants provided informed consent to participate in the study and for their data to be published. Participants were informed in advance of the purpose and the procedures of the study. Participants were assured of the confidentiality of their data. The experiments were carried out under established protocols and responsibility and commitment to the quality of the results obtained in this study is declared, ensuring that the procedures and methodologies used are in accordance with the relevant standards and regulations.

## Data availability statement

Data will be made available on request.

## CRediT authorship contribution statement

**Chong Shin Yee:** Writing – original draft, Formal analysis, Conceptualization. **Zul Ilham:** Supervision, Project administration, Conceptualization. **Acga Cheng:** Supervision, Project administration, Conceptualization. **Muhamad Hafiz Abd Rahim:** Formal analysis, Conceptualization. **Siti Hajar-Azhari:** Writing – original draft. **Mohd Hafis Yuswan:** Writing – original draft, Conceptualization. **Nurul Aqilah Mohd Zaini:** Writing – review & editing, Writing – original draft, Conceptualization. **Anna Reale:** Writing – original draft, Conceptualization. **Tiziana Di Renzo:** Writing – review & editing, Writing – original draft, Methodology, Data curation, Conceptualization. **Wan Abd Al Qadr Imad Wan-Mohtar:** Writing – review & editing, Writing – original draft, Supervision, Resources, Methodology, Investigation, Funding acquisition, Data curation, Conceptualization.

## Declaration of competing interest

The authors declare that the research was conducted in the absence of any commercial or financial relationships that could be construed as a potential conflict of interest.
