# Supplementary material for: Optimisation of fermentation conditions for the production of gamma-aminobutyric acid (GABA)-rich soy sauce
Source: Heliyon. 2024 Jun 18;10(13):e33147. doi: 10.1016/j.heliyon.2024.e33147 (PMC11261068; doi:10.1016/j.heliyon.2024.e33147)
Supplement: Multimedia component 1 [file mmc1.docx]

**SENSORY EVALUATION OF SOY SAUCE SAMPLES**

**Date:**

**Age: Gender:**

**INSTRUCTION**

Please evaluate the three (3) coded soy sauce samples presented to you.

The parameters to evaluate for each soy sauce sample are sweet, umami, salty, bitter, sour, astringent and overall acceptability. Taste the soy sauce provided into a plastic sauce plate by dipping the rice ball on it. Kindly take a bite of unsalted cracker as palate cleanser and rinse your mouth with the mineral water provided before tasting next sample.

Use the following number scores below to represent how much you like or dislike the soy sauce samples based on each parameter:

10-cm line scale assigned:

| 0 | 1 | 2 | 3 | 4 | 5 | 6 | 7 | 8 | 9 |
| --- | --- | --- | --- | --- | --- | --- | --- | --- | --- |
| None | Extreme weak | Very weak | Weak | Moderately weak | Neither weak nor strong | Moderately Strong | Strong | Very strong | Extremely strong |

| Samples | Taste | | | | | | Overall acceptability |
| --- | --- | --- | --- | --- | --- | --- | --- |
|  | Sweet | Umami | Salty | Bitter | Sour | Astringent |  |
| 525 |  |  |  |  |  |  |  |
| 701 |  |  |  |  |  |  |  |
| 815 |  |  |  |  |  |  |  |

Kindly leave your comment for improvement.

________________________________________________________________________________________________________________________________________________________________________________________________________________________________________________________________________________________________________________________________________________________________________
